# Improved home BP profile with dapagliflozin is associated with amelioration of albuminuria in Japanese patients with diabetic nephropathy: the Yokohama add-on inhibitory efficacy of dapagliflozin on albuminuria in Japanese patients with type 2 diabetes study (Y-AIDA study)

**DOI:** 10.1186/s12933-019-0912-3

**Published:** 2019-08-27

**Authors:** Sho Kinguchi, Hiromichi Wakui, Yuzuru Ito, Yoshinobu Kondo, Kengo Azushima, Uru Osada, Tadashi Yamakawa, Tamio Iwamoto, Jun Yutoh, Toshihiro Misumi, Kazutaka Aoki, Gen Yasuda, Taishi Yoshii, Takayuki Yamada, Syuji Ono, Tomoko Shibasaki-Kurita, Saho Hosokawa, Kazuki Orime, Masaaki Hanaoka, Hiroto Sasaki, Kohji Inazumi, Taku Yamada, Ryu Kobayashi, Kohji Ohki, Kotaro Haruhara, Yusuke Kobayashi, Takeharu Yamanaka, Yasuo Terauchi, Kouichi Tamura

**Affiliations:** 1 Department of Medical Science and Cardiorenal Medicine, Yokohama City University Graduate School of Medicine, 3-9 Fukuura, Kanazawa-ku, Yokohama, 236-0004 Japan; 2 Department of Endocrinology and Metabolism, Yokohama City University Graduate School of Medicine, 3-9 Fukuura, Kanazawa-ku, Yokohama, 236-0004 Japan; 3 Cardiovascular and Metabolic Disorders Program, Duke-NUS Medical School, Singapore, Singapore; 4 Department of Diabetes and Endocrinology, Saiseikai Yokohama South Hospital, Yokohama, Japan; 5 Department of Endocrinology and Diabetes, Yokohama City University Center Hospital, Yokohama, Japan; 6 Department of Nephrology and Hypertension, Saiseikai Yokohama South Hospital, Yokohama, Japan; 7 Department of Nephrology and Hypertension, Yokohama Minami Kyousai Hospital, Yokohama, Japan; 8 Department of Biostatistics and Epidemiology, Yokohama City University Graduate School of Medicine, Yokohama, Japan; 9 Department of Internal Medicine, Kanagawa Dental University, Yokosuka, Japan; 10 Department of Nephrology and Hypertension, Yokohama City University Center Hospital, Yokohama, Japan; 11 Department of Endocrinology and Metabolism, Yokohama Minami Kyousai Hospital, Yokohama, Japan; 12 Center for Novel and Exploratory Clinical Trials (Y-NEXT), Yokohama City University, Yokohama, Japan

**Keywords:** Type 2 diabetes mellitus, SGLT2 inhibitor, Blood pressure, Diabetic nephropathy, Albuminuria

## Abstract

**Background:**

The Y-AIDA study was designed to investigate the renal- and home blood pressure (BP)-modulating effects of add-on dapagliflozin treatment in Japanese individuals with type 2 diabetes mellitus (T2DM) and albuminuria.

**Methods:**

We conducted a prospective, multicenter, single-arm study. Eighty-six patients with T2DM, HbA1c 7.0–10.0%, estimated glomerular filtration rate (eGFR) ≥ 45 mL/min/1.73 m^2^, and urine albumin-to-creatinine ratio (UACR) ≥ 30 mg/g creatinine (gCr) were enrolled, and 85 of these patients were administered add-on dapagliflozin for 24 weeks. The primary and key secondary endpoints were change from baseline in the natural logarithm of UACR over 24 weeks and change in home BP profile at week 24.

**Results:**

Baseline median UACR was 181.5 mg/gCr (interquartile range 47.85, 638.0). Baseline morning, evening, and nocturnal home systolic/diastolic BP was 137.6/82.7 mmHg, 136.1/79.3 mmHg, and 125.4/74.1 mmHg, respectively. After 24 weeks, the logarithm of UACR decreased by 0.37 ± 0.73 (*P *< 0.001). In addition, changes in morning, evening, and nocturnal home BP from baseline were as follows: morning systolic/diastolic BP − 8.32 ± 11.42/− 4.18 ± 5.91 mmHg (both *P *< 0.001), evening systolic/diastolic BP − 9.57 ± 12.08/− 4.48 ± 6.45 mmHg (both *P *< 0.001), and nocturnal systolic/diastolic BP − 2.38 ± 7.82/− 1.17 ± 5.39 mmHg (*P *= 0.0079 for systolic BP, *P *= 0.0415 for diastolic BP). Furthermore, the reduction in UACR after 24 weeks significantly correlated with an improvement in home BP profile, but not with changes in other variables, including office BP. Multivariate linear regression analysis also revealed that the change in morning home systolic BP was a significant contributor to the change in log-UACR.

**Conclusions:**

In Japanese patients with T2DM and diabetic nephropathy, dapagliflozin significantly improved albuminuria levels and the home BP profile. Improved morning home systolic BP was associated with albuminuria reduction.

*Trial registration* The study is registered at the UMIN Clinical Trials Registry (UMIN000018930; http://www.umin.ac.jp/ctr/index-j.htm). The study was conducted from July 1, 2015 to August 1, 2018.

**Electronic supplementary material:**

The online version of this article (10.1186/s12933-019-0912-3) contains supplementary material, which is available to authorized users.

## Background

The number of patients with type 2 diabetes mellitus (T2DM) is increasing, and renal and cardiovascular complications often provoke serious conditions in diabetic patients [1–3]. Albuminuria is an important risk factor in the exacerbation of diabetic nephropathy (DN) and onset of cardiovascular diseases (CVD) in patients with T2DM [4–6]. Moreover, remission of albuminuria was associated with reduction in the risk of CVD [5]. Therefore, albuminuria is an important therapeutic target for renal and cardiovascular complications in patients with T2DM. Appropriate blood pressure (BP) management is important to suppress albuminuria [7]. Current guidelines also recommend the intensive control of BP as well as blood glucose to reduce albuminuria and prevent the development of DN [8].

Dapagliflozin is a sodium–glucose cotransporter-2 (SGLT2) inhibitor that primarily acts by increasing the excretion of glucose in urine to decrease the blood glucose level. Several large randomized trials have demonstrated that SGLT2 inhibitors reduce the occurrence of CVD and have renal protective effects in T2DM patients [9–13]. Furthermore, office BP-lowering effects of SGLT2 inhibitors have been demonstrated in these large randomized trials and in other studies [9, 11, 14, 15]. However, subjects in these trials included patients without DN, and few Asian patients were enrolled. In addition, no reports to date have investigated whether SGLT2 inhibitors could lower out-of-office BP in patients with T2DM and DN.

Compared with office BP, out-of-office BP, such as home BP measurements and ambulatory BP monitoring (ABPM), is a better predictor of CVD events, exacerbation of chronic kidney disease (CKD), transition to end-stage renal disease (ESRD), and mortality [16–19]. In the current guidelines, home BP monitoring is recommended for appropriate BP control in patients with T2DM [8]. In addition, both average home BP levels and parameters of BP variability obtained from home BP measurements have been reported as associated with organ damage and CVD events [20]. Furthermore, in patients with T2DM, day-by-day variability in home BP was associated with an increase in urinary albumin excretion, independent of other risk factors [21, 22]. Therefore, improved control of average home BP levels to avoid variability in addition to standard office BP management may represent a potential therapeutic strategy for patients with T2DM.

In the present study, we investigated the renal effects of 24-week add-on dapagliflozin treatment on urinary albumin excretion in Japanese patients with T2DM and DN. We also examined the effects of dapagliflozin on home BP profiles, including average home BP levels and home BP variability.

## Methods

This study complied with the ethical principles of the Declaration of Helsinki, and was approved by the institutional ethics committee at each participating hospital. The study is registered at the UMIN Clinical Trials Registry (UMIN000018930; http://www.umin.ac.jp/ctr/index-j.htm). All patients provided written informed consent prior to participation. Registration and collected data were audited by an independent data management center.

### Study design

A 24-week prospective, multicenter, single-arm study was conducted from July 1, 2015 to August 1, 2018 at four hospitals in Japan: Yokohama City University Hospital, Yokohama City University Medical Center, Saiseikai Yokohama South Hospital, and Yokohama Minami Kyousai Hospital.

This study consisted of a 14-week run-in period and 24-week add-on dapagliflozin therapy period. Eligible individuals were aged between 20 and < 80 years old with inadequately controlled type 2 diabetes (HbA1c 7.0–10.0%) and estimated glomerular filtration (eGFR) ≥ 45 mL/min/1.73 m^2^, based on the revised equation for the Japanese population [23]. To meet the criterion for renal dysfunction, eligible individuals had a urinary albumin-to-creatinine ratio (UACR) ≥ 30 mg/g creatinine (gCr) at screening. The exclusion criteria were as follows: (i) women who were pregnant or breastfeeding; (ii) significantly elevated creatinine kinase (CK) level (CK > 765 U/L); (iii) significantly abnormal liver function (aspartate aminotransferase (AST) > 96 U/L and/or alanine aminotransferase (ALT) > 135 U/L); (iv) New York Heart Association class IV congestive heart failure or acute congestive heart failure; (v) history of diabetic ketoacidosis, diabetic coma, or pre coma within the 6 months prior to screening; (vi) severe infection; (vii) pre or post-surgery; (viii) serious trauma; (ix) known hypersensitivity to dapagliflozin; (x) on-going treatment with SGLT2 inhibitors or a history of treatment with SGLT2 inhibitors in the 1 month prior to screening; and (xi) patients judged by the investigator to be ineligible for some other reason.

After the run-in period, eligible individuals were initially given dapagliflozin 5 mg as add-on to existing therapy in the morning (visit of 0 weeks), and the dose of dapagliflozin could be titrated up to 10 mg daily 8 weeks or 16 weeks after treatment initiation to achieve the HbA1c target (< 7.0%) if necessary. In the study period, anti-diabetic, anti-hypertensive, and anti-dyslipidemia medication regimens were not modified in principle.

### Physical findings

Body weight, body mass index (BMI), office BP, and pulse rate (PR) were measured at baseline (0 weeks) and after a period of 8 weeks, 16 weeks, and 24 weeks of treatment.

### Home BP and day-by-day home BP variability

All participants took home BP readings using a validated and internet-interfaced BP monitor (HEM-7252G-HP, Omron Corporation, Kyoto) using a mobile telecommunication system (MedicalLINK), which enabled automatic transmission of all home BP readings to a server for data recording after each measurement to allow accurate analysis of the home BP profile [24, 25]. Three home BP readings were taken at 1-min intervals in a sitting position in the morning and the evening for a 7-day study period before the 0 week and 24 week visits. Morning BP was measured before breakfast within 1 h of waking and before taking anti-hypertensive medication. Evening BP was measured before going to bed. The mean morning and evening home BPs were defined as the average of BPs in the morning and evening for the 7-day study period, and were the mean of 3 readings in the morning and evening each day. Morning and evening day-by-day home BP variabilities were defined as the standard deviation (SD) of mean morning and evening home BPs each day for the 7-day study period, as described previously [26].

### Nocturnal home BP and nocturnal home BP variability

The HEM-7252G-HP monitor can record BP readings at fixed times, and was preset to record nocturnal home BP measurements at 2:00, 3:00, 4:00, and 5:00 a.m. (4 time points). Participants were instructed to measure their nocturnal home BP for a 7-day study period before the 0- and 24-week visits. Mean nocturnal home BP was defined as the average nighttime BP for the 7-day study period, which represented the mean of 4 readings each night. Nocturnal home BP variability was defined as the SD of mean nocturnal home BP each day for the 7-day study period.

### Circadian BP pattern

Based on home BP measurements, circadian BP patterns were classified as follows: non-dipper, dipper, extreme-dipper, or riser pattern. The nocturnal BP decline was defined by calculating the percentage of the decline in the systolic BP during the nighttime. Non-dippers were defined as having nocturnal declines that were 0–10% of the daytime systolic BP. Dippers were defined as having nocturnal declines that were 10–20% of the daytime systolic BP. Extreme-dippers were defined as having nocturnal declines that were > 20% of the daytime systolic BP. Risers were defined as having nocturnal declines that were < 0% of the daytime systolic BP [27]. Daytime systolic BP in home BP measurement was defined as the average of the mean morning and evening home systolic BP. Nighttime systolic BP in home BP measurement was defined as the mean nocturnal home systolic BP.

### Laboratory measurements

Blood and urine sampling was performed in a fasted state at baseline (0 weeks) and after 8 weeks, 16 weeks, and 24 weeks of treatment. Urinalysis was performed in spot urine samples at the applicable visits. Measurement methods were as follows. UACR were determined using a turbidimetric immunoassay (SRL laboratory, Tokyo, Japan). Insurance-uncovered laboratory parameters (urine sodium-to-creatinine ratio, urine liver-type fatty-acid binding protein (L-FABP)-to-creatinine ratio, urine type IV collagen-to-creatinine ratio, urine 8-hydroxy-2′-deoxyguanosine (8-OHdG)-to-creatinine ratio, low-density lipoprotein (LDL)-cholesterol, pentosidine) were also measured by SRL Inc. (Tokyo, Japan) as described previously [28–30]. We performed diacron-reactive oxygen metabolites (d-ROMs) test and biological antioxidant potential (BAP) test at a laboratory in Yokohama city university, in order to evaluate oxidative stress and reduction power [31]. Other laboratory parameters were determined using routine methods in the department of clinical chemistry in each hospital.

### Endpoints and safety

The primary endpoint was the change in the natural logarithm of the UACR (log-UACR) of the spot urine sample from baseline to 24 weeks. Subgroup analysis was also conducted to determine the change from baseline in the log-UACR at week 24 using angiotensin-converting enzyme (ACE) inhibitors or angiotensin receptor blockers (ARBs) at baseline. Additionally, to interpret the extent of changes in albuminuria for their clinical relevance, the change in the non-logarithmic UACR values were analyzed. Changes from baseline in other measurements, including home BP parameters, were secondary endpoints. Based on home BP measurements, circadian BP patterns were classified at baseline and week 24. Safety and tolerability were assessed based on adverse event (AE) reported throughout the study period. In addition, drug adherence was assessed by the proportion of patients taking dapagliflozin on the visit day during the study period.

### Statistical analysis

The primary analysis was the estimation of changes in the natural logarithm of the UACR after per-protocol dapagliflozin treatment for 24 weeks in a single arm using a one-sided paired t-test. Based on a previous study of dapagliflozin [32], we assumed that the natural logarithm of the UACR would be normally distributed, that the change in the natural logarithm of the UACR from baseline to 24 weeks following dapagliflozin treatment would be − 0.5, and that the SD of the primary endpoint would be 1.7. To detect a difference in the primary endpoint in a single arm with a one-sided alpha error of 5% and power of 80%, 72 participants were therefore required.

Data in the text and tables are presented as the mean, mean ± SD, percentage, or median (interquartile range). Data in the figures are presented as the mean ± standard error (SE). For the statistical analysis of the difference from baseline to each visit, the change in each parameter after add-on dapagliflozin therapy for 8, 16, or 24 weeks was analyzed using a one-sided paired *t*-test. Associations between the primary endpoint and other variables were assessed using Pearson’s correlation coefficients. Multivariate linear regression analyses were performed to identify the factors affecting the change in log-UACR. The independent variables entered into the model for multivariate regression analysis were as follows: diabetes duration, baseline values of fasting blood sugar (FBS), glycated hemoglobin (HbA1c), and white blood cell (WBC) levels, and changes from baseline in the morning home systolic BP, office systolic BP, HbA1c, body weight, and diacron-reactive oxygen metabolites (d-ROMs) at week 24, which were significantly different or showed a decreasing tendency, after 24 weeks of dapagliflozin treatment. Frequency differences in the circadian BP patterns between baseline and week 24 were assessed using the Chi-squared test. Statistical analysis was performed using SAS version 9.4 (SAS Institute Japan) at the department of biostatistics and epidemiology in Yokohama city university graduate school of medicine, and a value of *P* < 0.05 was considered statistically significant.

## Results

### Baseline patient characteristics

A total of 86 patients with T2DM and albuminuria were enrolled, and 85 (65 males and 20 females) received study medication. One participant withdrew consent to participate before receiving study medication. Table 1 shows the baseline characteristics of the 85 participants. The mean age of participants was 65.1 years, mean duration of T2DM was 12.6 years, mean HbA1c was 7.83%, and mean BMI was 27.05 kg/m^2^. The median UACR was 181.5 mg/gCr (interquartile range 47.85, 638.0) with mean eGFR 67.34 mL/min/1.73 m^2^. Most participants had hypertension (89.2%), and mean office systolic/diastolic BP was 142.2/79.0 mmHg; 68.2% of patients were taking angiotensin II receptor blockers (ARBs) and 4.7% were taking angiotensin-converting enzyme (ACE) inhibitors (Table 1).

**Table 1:** Baseline characteristics

Variables	Mean ± SD or %
Age (year)	65.1 ± 10.1
Sex (male/female)	65/20
Body mass index (kg/m^2^)	27.1 ± 4.7
Diabetes duration (year)	12.6 ± 9.2
Current smoker n (%)	17 (20.0)
Past smoker n (%)	32 (37.6)
Current drinker n (%)	43 (50.6)
Past drinker n (%)	12 (14.1)
Hypertension n (%)	74 (89.2)
Dyslipidemia n (%)	72 (86.7)
Hyperuricemia n (%)	19 (22.9)
Previous cardiovascular disease n (%)	21 (24.7)
Office blood pressure	
SBP (mmHg)	142.2 ± 18.9
DBP (mmHg)	79.0 ± 12.0
Glucose metabolism	
Fasting plasma glucose (mg/dL)	156.9 ± 38.4
HbA1c (%)	7.8 ± 0.7
Renal function	
Serum creatinine (mg/dL)	0.9 ± 0.2
eGFR (mL/min/1.73 m^2^)	67.3 ± 17.6
Median UACR (mg/gCr) (interquartile range)	181.5 (47.9, 638.0)
Lipid metabolism	
Total cholesterol (mg/dL)	184.4 ± 37.5
LDL cholesterol (mg/dL)	102.0 ± 29.0
HDL cholesterol (mg/dL)	52.7 ± 15.4
Triglyceride (mg/dL)	173.8 ± 115.1
Antidiabetic agents	
Insulin n (%)	28 (37.8)
Biguanides n (%)	49 (66.2)
DPP-4 inhibitors n (%)	47 (63.5)
Sulfonylureas n (%)	23 (31.1)
α-Glucosidase inhibitors n (%)	17 (23.0)
Thiazolidinediones n (%)	12 (16.2)
Glinides n (%)	7 (9.5)
GLP1 agonists n (%)	8 (10.8)
Antihypertensive agents	
RAS inhibitors	
Angiotensin II receptor blockers n (%)	58 (68.2)
Angiotensin-converting enzyme inhibitors n (%)	4 (4.7)
Calcium-channel blockers n (%)	52 (61.2)
Diuretics n (%)	10 (11.8)
α1-Blockers n (%)	2 (2.4)
β-Blockers n (%)	7 (8.2)
Spironolactone n (%)	3 (3.5)
α2-Agonists n (%)	2 (2.4)
Antihyperlipidemic agents	
Statins n (%)	55 (64.7)
Fibrates n (%)	4 (4.7)
Antihyperuricemic agents n (%)	16 (18.8)

Values are mean ± standard deviation (SD)

*SBP* systolic blood pressure, *DBP* diastolic blood pressure, *HbA1c* glycated hemoglobin, *eGFR* estimated glomerular filtration rate, *UACR* urine albumin-to-creatinine ratio, *LDL* low-density lipoprotein, *HDL* high-density lipoprotein, *DPP-4* dipeptidyl peptidase 4, *GLP1* glucagon-like peptide 1, *RAS* renin-angiotensin system

### Effects of add-on dapagliflozin therapy on renal endpoints

**Fig. 1:**
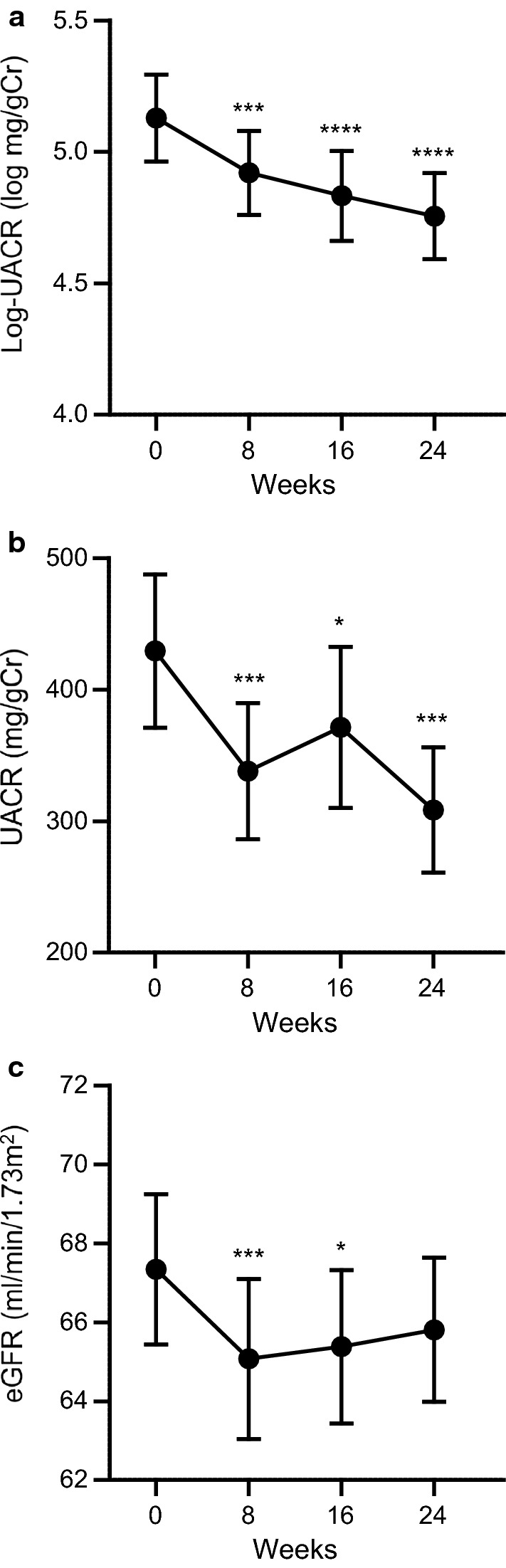
**a** Natural logarithm of urine albumin-to-creatinine ratio (log-UACR), **b** non-logarithmic UACR values, and **c** estimated glomerular filtration (eGFR) induced by add-on dapagliflozin treatment during the 24-week study period. The change from baseline to week 24 was analyzed using a one-sided paired *t*-test. *P < 0.05, ***P < 0.005, ****P < 0.001

At 8 weeks, a significant reduction in the natural logarithm of UACR (log-UACR) from baseline was noted, with a difference from baseline of 0.23 ± 0.72 (*P *= 0.0026) (Fig. 1a). Log-UACR subsequently declined gradually, and there was a further reduction in log-UACR from baseline at 24 weeks (0.37 ± 0.73, *P *< 0.001) (Fig. 1a). The decrease in non-logarithmic values of UACR from baseline was 109.95 ± 333.04 mg/gCr at week 24 (*P *= 0.0017) (Fig. 1b). The combination therapy of SGLT2 inhibitors and ARBs/ACE inhibitors is expected to have greater renoprotective effects compared with administration of either drug alone because of synergistic effects in patients with DN [33]. In this study, however, there was no significant difference in the reduction in log-UACR from baseline at 24 weeks between in patients receiving ARBs/ACE inhibitors (− 0.37 ± 0.81, *P *= 0.0005) and in patients who were not receiving ARBs/ACE inhibitors (− 0.36 ± 0.53, *P *= 0.0010; the difference in the mean values between patients receiving ARBs/ACE inhibitors and patients not receiving ARBs/ACE inhibitors [95% confidence interval] was 0.00 [− 0.35; 0.35]). On the other hand, although treatment with add-on dapagliflozin initially decreased eGFR at week 8 (− 2.27 ± 7.92 mL/min/1.73 m^2^, *P *= 0.0049), continuous treatment with dapagliflozin resulted in a gradual recovery of eGFR afterwards and change in eGFR from baseline to week 24 was not statistically significant (− 1.53 ± 8.58 mL/min/1.73 m^2^, *P *= 0.0518) (Fig. 1c).

### Effects of add-on dapagliflozin therapy on physical findings, markers of arterial stiffness, glucose and lipid metabolism, liver function, and pro-inflammatory markers

From baseline to week 8, add-on dapagliflozin therapy significantly decreased office systolic BP (− 4.4 ± 17.7 mmHg, *P *= 0.0163) (Table 2). This decrease in office systolic BP was maintained at week 16 and week 24. Office diastolic BP significantly decreased by 2.3 ± 11.0 mmHg (*P *= 0.0354) at week 24. Add-on dapagliflozin therapy for 24 weeks did not change the pulse rate, but significantly reduced body weight and BMI throughout the treatment period.

**Table 2:** Effects of add-on dapagliflozin therapy on physical findings, arterial stiffness, glycolipid metabolism, liver function, and pro-inflammatory markers

	Baseline	Week 8	Week 16	Week 24
BW (kg)	73.9 ± 16.6	72.5 ± 16.5****	72.2 ± 16.6****	72.1 ± 16.6****
BMI (kg/m^2^)	27.1 ± 4.5	26.6 ± 4.5****	26.6 ± 4.6****	26.5 ± 4.6****
Office				
SBP (mmHg)	142.2 ± 18.9	137.6 ± 18.4*	136.8 ± 18.3*	137.3 ± 19.0*
DBP (mmHg)	79.0 ± 12.0	77.7 ± 12.7	77.3 ± 11.3	75.9 ± 11.4*
PR (bpm)	82.9 ± 14.2	82.6 ± 14.5	82.0 ± 13.0	81.1 ± 12.7
PP (mmHg)	63.2 ± 18.2	59.9 ± 15.6*	59.5 ± 17.0*	61.4 ± 19.0
MAP (mmHg)	100.0 ± 11.8	97.6 ± 12.9	97.1 ± 11.5*	96.3 ± 11.3*
Double product (bpm × mmHg)	11,949.9 ± 2862.8	11,431.8 ± 2648.7^*^	11,309.1 ± 2493.0*	11,169.4 ± 2534.7**
FBS (mg/dL)	156.9 ± 38.4	143.3 ± 40.9***	142.0 ± 36.9***	141.1 ± 42.9***
HbA1c (%)	7.8 ± 0.7	7.4 ± 0.7****	7.4 ± 0.7****	7.3 ± 0.7****
LDL (mg/dL)	102.0 ± 29.0	N/A	N/A	102.4 ± 31.0
HDL (mg/dL)	52.7 ± 15.4	N/A	N/A	54.1 ± 14.9
T. chol (mg/dL)	184.4 ± 37.5	N/A	N/A	185.0 ± 35.8
TG (mg/dL)	173.8 ± 115.1	N/A	N/A	177.5 ± 130.4
AST (U/L)	28.3 ± 12.8	25.7 ± 8.9***	24.8 ± 8.5****	24.1 ± 8.1****
ALT (U/L)	33.4 ± 19.5	28.2 ± 14.8****	27.1 ± 16.2****	25.7 ± 14.0****
ALP (U/L)	235.0 ± 77.4	226.6 ± 73.5**	221.4 ± 71.6****	221.5 ± 69.2****
γGTP (U/L)	47.4 ± 34.5	39.4 ± 28.6****	38.5 ± 29.0****	37.0 ± 25.2****
WBC (/mm^3^)	7289.8 ± 1930.2	7468.1 ± 1991.5	7339.3 ± 1767.2	7182.5 ± 1699.9
Plt (× 10^4^/mm^3^)	22.4 ± 6.4	22.7 ± 6.6	22.6 ± 6.5	22.3 ± 6.5

Values are mean ± standard deviation (SD)

*BW* body weight, *BMI* body mass index, *SBP* systolic blood pressure, *DBP* diastolic blood pressure, *PR* pulse rate, *PP* pulse pressure, *MAP* mean arterial pressure, *bpm* beat per minute, *FBS* fasting blood sugar, *HbA1c* glycated hemoglobin, *LDL* low-density lipoprotein, *HDL* high-density lipoprotein, *T. chol* total cholesterol, *TG* triglyceride, *AST* aspartate aminotransferase, *ALT* alanine aminotransferase, *ALP* alkaline phosphatase, *γ-GTP* γ-glutamyl transpeptidase, *WBC* white blood cell, *Plt* platelet, *N/A* not available

*P < 0.05, **P < 0.01, ***P < 0.005, ****P < 0.001 versus baseline

Pulse pressure (PP: difference between systolic BP and diastolic BP), mean arterial pressure (MAP: 2/3 diastolic BP + 1/3 systolic BP), and double product (heart rate × systolic BP) are markers of arterial stiffness that have been linked to cardiovascular outcomes [34, 35]. Add-on dapagliflozin treatment significantly decreased PP at week 8 (− 3.4 ± 15.3 mmHg, *P *= 0.0284) and week 16 (− 3.7 ± 18.0 mmHg, *P *= 0.0372), but not at week 24 (−2.0 ± 18.3 mmHg, *P *= 0.1745) (Table 2). MAP also decreased by 2.430 ± 11.371 mmHg (*P *= 0.0332) at week 16 and 2.995 ± 11.988 mmHg (*P *= 0.0168) at week 24. Furthermore, double product declined by 425.5 ± 1836.5 (*P *= 0.0321) at week 8, and the improvement in double product was maintained at week 16 and week 24.

Treatment with add-on dapagliflozin resulted in a significant decrease in fasting blood sugar (FBS) and HbA1c at 8 weeks, and this improvement in glucose metabolism was maintained throughout the study period (Table 2). Similarly, parameters of liver function were significantly improved at week 8, and the improvement was sustained at 16 and 24 weeks (Table 2).

Sub-clinical inflammation was reported to be involved in the pathogenesis of endothelial dysfunction, which leads to vascular complications including nephropathy in diabetic patients [36]. However, add-on dapagliflozin therapy for 24 weeks did not change the pro-inflammatory markers, such as WBC and platelets (Plt) in the present study (Table 2).

### Effects of add-on dapagliflozin therapy on home BP profile

**Fig. 2:**
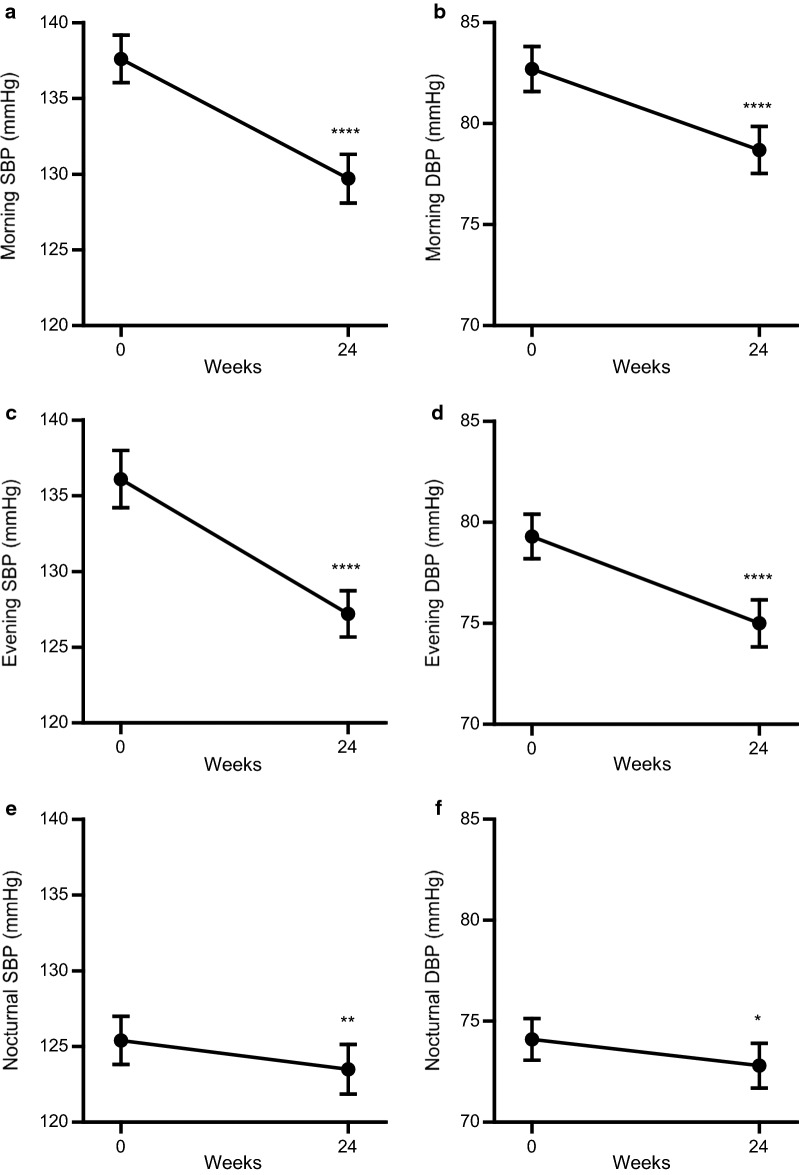
**a** Morning home systolic blood pressure (SBP), **b** morning home diastolic blood pressure (DBP), **c** evening home SBP, **d** evening home DBP, **e** nocturnal home SBP, and **f** nocturnal home DBP at baseline and after 24 weeks of add-on dapagliflozin treatment (week 24). The change from baseline to week 24 was analyzed using a one-sided paired *t*-test. *P < 0.05, **P < 0.01, ****P < 0.001

Baseline morning, evening, and nocturnal home systolic/diastolic BP were 137.6/82.7 mmHg, 136.1/79.3 mmHg, and 125.4/74.1 mmHg, respectively. After 24 weeks of dapagliflozin treatment, changes in morning, evening, and nocturnal home BP from baseline were morning systolic/diastolic BP − 8.32 ± 11.42/− 4.18 ± 5.91 mmHg (both *P *< 0.001), evening systolic/diastolic BP − 9.57 ± 12.08/− 4.48 ± 6.45 mmHg (both *P *< 0.001), and nocturnal systolic/diastolic BP − 2.38 ± 7.82/− 1.17 ± 5.39 mmHg (*P *= 0.0079 for systolic BP, *P *= 0.0415 for diastolic BP) without an evident increase in PR (Table 3, Fig. 2). In addition, with respect to day-by-day home BP variability; systolic day-by-day BP variability in the morning, evening, and nocturnal period and diastolic day-by-day BP variability in the nocturnal period were significantly decreased at week 24 (Table 3).

**Table 3:** Effects of add-on dapagliflozin therapy on home blood pressure profile

	Baseline	Week 24	*P* value
Morning			
SBP (mmHg)	137.6 ± 14.5	129.7 ± 14.8	*P *< 0.001
SBP-SD (mmHg)	8.8 ± 4.7	7.9 ± 3.8	*P* = 0.0496
DBP (mmHg)	82.7 ± 10.3	78.7 ± 10.7	*P *< 0.001
DBP-SD (mmHg)	5.2 ± 3.4	4.9 ± 3.0	*P* = 0.1494
PR (beats/min)	72.9 ± 10.6	71.4 ± 10.8	*P* = 0.0785
Evening			
SBP (mmHg)	136.1 ± 17.4	127.2 ± 14.1	*P *< 0.001
SBP-SD (mmHg)	9.4 ± 5.0	8.4 ± 4.0	*P* = 0.0143
DBP (mmHg)	79.3 ± 10.1	75.0 ± 10.7	*P *< 0.001
DBP-SD (mmHg)	5.7 ± 3.1	5.0 ± 2.5	*P* = 0.1331
PR (beats/min)	76.5 ± 11.3	75.1 ± 11.0	*P* = 0.0414
Nocturnal			
SBP (mmHg)	125.4 ± 14.7	123.5 ± 15.1	*P* = 0.0079
SBP-SD (mmHg)	8.9 ± 4.2	7.3 ± 3.2	*P* = 0.0049
DBP (mmHg)	74.1 ± 9.5	72.8 ± 10.2	*P* = 0.0415
DBP-SD (mmHg)	5.6 ± 2.8	4.7 ± 2.2	*P* = 0.0092
PR (beats/min)	67.6 ± 11.2	65.6 ± 10.3	*P* = 0.0260

Values are mean ± standard deviation (SD)

*SBP* systolic blood pressure, *DBP* diastolic blood pressure, *PR* pulse rate

### Effects of add-on dapagliflozin therapy on circadian BP patterns

**Fig. 3:**
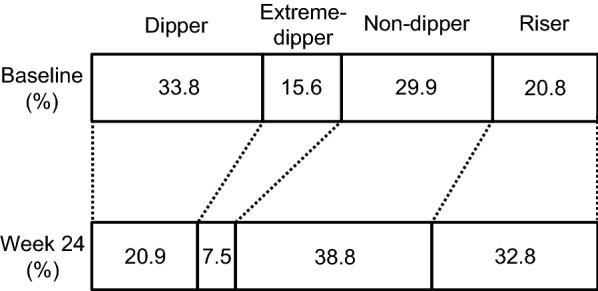
The distribution of circadian blood pressure patterns at baseline and after 24 weeks of add-on dapagliflozin treatment (week 24)

Based on home BP measurements, circadian BP patterns were classified at baseline and week 24. The results of home BP measurement revealed that 33.8% of patients had the dipper pattern, 15.6% of patients had the extreme-dipper pattern, 29.9% of patients had the non-dipper pattern, and 20.8% of patients had the riser pattern at baseline (Fig. 3). After add-on dapagliflozin treatment for 24 weeks, 20.9% of patients had the dipper pattern, 7.5% of patients had the extreme-dipper pattern, 38.8% of patients had the non-dipper pattern, and 32.8% of patients had the riser pattern (Fig. 3). The proportion of dippers and extreme-dippers was decreased, and the proportion of non-dippers and risers was increased (*P *= 0.0102).

### Effects of add-on dapagliflozin therapy on urine and systemic oxidative stress markers

**Fig. 4:**
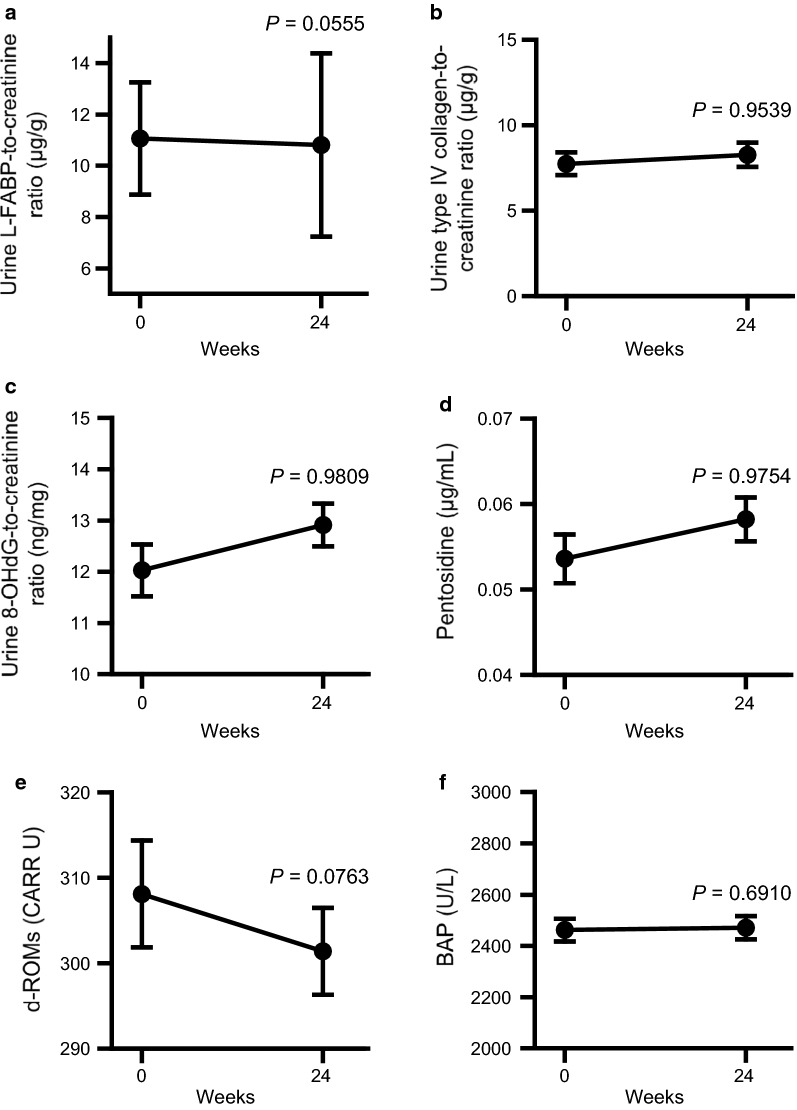
**a** Urine liver-type fatty-acid binding protein (L-FABP)-to-creatinine ratio, **b** urine type IV collagen-to-creatinine ratio, **c** urine 8-hydroxy-2′-deoxyguanosine (8-OHdG)-to-creatinine ratio, **d** pentosidine, **e** diacron-reactive oxygen metabolites (d-ROMs), and **f** biological antioxidant potential (BAP) at baseline and after 24 weeks of add-on dapagliflozin treatment (week 24). Change from baseline to week 24 was analyzed using a one-sided paired *t*-test

Add-on dapagliflozin treatment tended to reduce the urinary liver-type fatty-acid binding protein (L-FABP)-to-creatinine ratio (− 0.121 ± 0.686, *P *= 0.0555) and diacron-reactive oxygen metabolites (d-ROMs) (− 6.3 ± 39.4, *P *= 0.0763) at week 24 (Fig. 4). Urine type IV collagen-to-creatinine ratio, urine 8-hydroxy-2′-deoxyguanosine (8-OhdG)-to-creatinine ratio, pentosidine, and biological antioxidant potential (BAP) did not change between baseline and week 24.

### Assessment of factors contributing to improvement in albuminuria

**Fig. 5:**
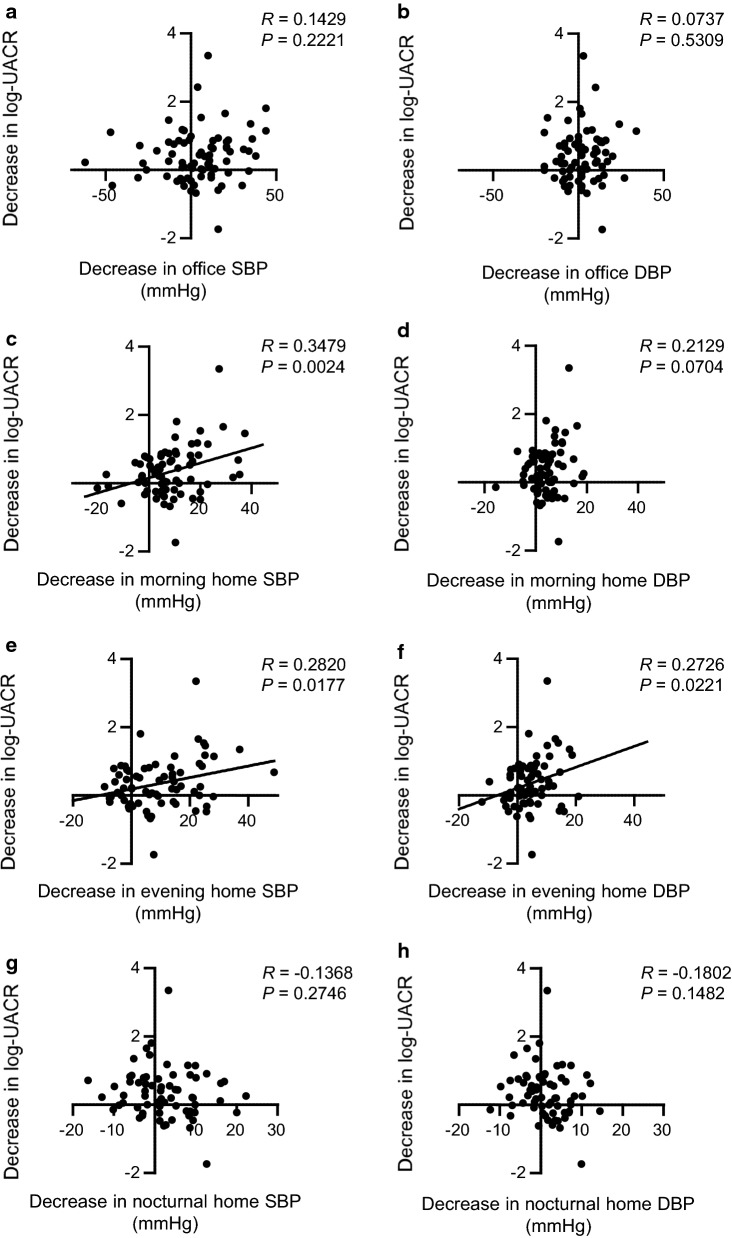
Univariate correlation analysis between the decreases in office systolic blood pressure (SBP) and diastolic blood pressure (DBP), morning home SBP and DBP, evening home SBP and DBP, and nocturnal home SBP and DBP, and the decrease in urine albumin-to-creatinine ratio (UACR)

The univariate correlation analysis identified significant positive relationships between decreased morning home systolic BP and evening home systolic/diastolic BP, and reduction in log-UACR (morning home systolic BP: *R *= 0.3479, *P *= 0.0024; evening home systolic BP: *R *= 0.2820, *P *= 0.0177; evening home diastolic BP: *R *= 0.2726, *P *= 0.0221) (Fig. 5). However, no such relationship was identified between decreased office systolic/diastolic BP, body weight, BMI, FBS, HbA1c, WBC, and Plt, and the reduction in log-UACR (Fig. 5, Additional file 1: Figure S1). Additionally, to examine whether an improvement in the home BP profile contributed to the reduction in log-UACR, we performed the multivariate linear regression analysis of the independent variables including the changes in morning home systolic BP, office systolic BP, weight, HbA1c, and d-ROMs (an oxidative stress marker), which were significantly different or tended to decrease after 24 weeks of dapagliflozin treatment. As shown in Table 4, the results of the multivariate linear regression analysis indicated that the change in the morning home systolic BP was a significant contributor to the changes in log-UACR. In another model, although we added change in the evening home systolic or diastolic BP instead of the morning home systolic BP with variables of the model, no significant association between the changes in log-UACR and variables, including the changes in evening home BPs, was observed (data not shown).

**Table 4:** Multivariate linear regression analysis of factors associated with the change in log-UACR

	B	SE	β	*P* value
Change in morning home SBP (mmHg)	0.01626	0.00733	0.26330	0.0305
Diabetes duration (year)	− 0.02156	0.01236	− 0.24438	0.0867
Fasting blood sugar (mg/dL)	− 0.00332	0.00219	− 0.17868	0.1353
Change in office SBP (mmHg)	0.00668	0.00482	0.17351	0.1712
HbA1c (%)	− 0.22415	0.16215	− 0.18777	0.1723
WBC (/mm^3^)	0.00005302	0.00005094	0.14462	0.3025
Change in body weight (kg)	0.04017	0.04919	0.11133	0.4176
Change in HbA1c (%)	− 0.04314	0.16195	− 0.03561	0.7909
Change in d-ROMs (CARR U)	0.00057766	0.00244	0.03157	0.8141

Adjusted R^2^ = 0.1942, *P* = 0.01. Variables included in the model were changes in morning home SBP, office SBP, body weight, HbA1c, and d-ROMs, and baseline values of diabetes duration, fasting blood sugar, HbA1c, and WBC

*B* regression coefficient, *SE* standard error, *β* standardized regression coefficient, *SBP* systolic blood pressure, *HbA1c* glycated hemoglobin, *WBC* white blood cell, *d-ROMs* diacron-reactive oxygen metabolites

### Safety and tolerability

Eight subjects (9.5%) experienced mild or moderate episodes of hypoglycemia (Table 5). Other AEs reported throughout the study period occurred at a frequency < 3%, or may not have been related to dapagliflozin treatment. No AEs leading to discontinuation were reported. The proportion of patients taking dapagliflozin on the visit day during the study period was 99.6 ± 1.0%. Add-on dapagliflozin treatment was therefore well tolerated, and patients showed good treatment adherence.

**Table 5:** Adverse events

Event	Grade 1	Grade 2	Grade 3
Special interest categories			
Hypoglycemia	6 (7.1%)	2 (2.4%)	0 (0.0%)
Volume depletion	2 (2.4%)	0 (0.0%)	0 (0.0%)
Urinary tract/genital infection	0 (0.0%)	1 (1.2%)	0 (0.0%)
Cutaneous symptom	0 (0.0%)	0 (0.0%)	0 (0.0%)
Others			
Cold	2 (2.4%)	4 (4.7%)	0 (0.0%)
Influenza	0 (0.0%)	3 (3.5%)	0 (0.0%)
Pollenosis	1 (1.2%)	2 (2.4%)	0 (0.0%)
Hypertension	0 (0.0%)	2 (2.4%)	0 (0.0%)
Angina pectoris	0 (0.0%)	0 (0.0%)	1 (1.2%)
Cataract	0 (0.0%)	0 (0.0%)	1 (1.2%)
Retinal detachment	0 (0.0%)	0 (0.0%)	1 (1.2%)
Vitreous surgery due to retinopathy	0 (0.0%)	0 (0.0%)	1 (1.2%)
Cough variant asthma	0 (0.0%)	1 (1.2%)	0 (0.0%)
Elevated LDL	0 (0.0%)	1 (1.2%)	0 (0.0%)
Fever	0 (0.0%)	1 (1.2%)	0 (0.0%)
Fracture	0 (0.0%)	1 (1.2%)	0 (0.0%)
Hemorrhagic stool	0 (0.0%)	1 (1.2%)	0 (0.0%)
Hordeolum	0 (0.0%)	1 (1.2%)	0 (0.0%)
Prostatitis	0 (0.0%)	1 (1.2%)	0 (0.0%)
Elevated CK	1 (1.2%)	0 (0.0%)	0 (0.0%)
Nausea	1 (1.2%)	0 (0.0%)	0 (0.0%)
Odontectomy	1 (1.2%)	0 (0.0%)	0 (0.0%)
Pruritus	1 (1.2%)	0 (0.0%)	0 (0.0%)
Thamuria	1 (1.2%)	0 (0.0%)	0 (0.0%)
Wobbliness	1 (1.2%)	0 (0.0%)	0 (0.0%)

Values are n (%)

Evaluation of adverse events (AEs) was performed based on Common Terminology Criteria for Adverse Event version 4.0. Grade 1: mild (no need for therapeutic intervention); Grade 2: moderate (needs therapeutic intervention in outpatient clinic); Grade 3: severe (needs therapeutic intervention by admission); Grade 4: life threatening, or places the participant, in the view of the investigator, at immediate risk of death from the experience as it occurred; Grade 5: death. AEs of Grade 4 or 5 were not reported

*LDL* low-density lipoprotein cholesterol, *CK* creatine kinase

## Discussion

The main finding of this prospective, multicenter, interventional, single-arm study was that add-on dapagliflozin therapy for 24 weeks reduced albuminuria without decreasing eGFR in Japanese patients with T2DM and early-stage DN (eGFR ≥ 45 mL/min/1.73 m^2^ and UACR ≥ 30 mg/gCr), concomitant with improvement in home BP profile. Three new findings were obtained in this study: (i) dapagliflozin decreased out-of-office BP, which contributed to the ameliorating effects of dapagliflozin on albuminuria; (ii) dapagliflozin improved BP variability, which is an indicator of BP quality control; and (iii) Japanese patients with T2DM and DN, who have high salt sensitivity genetically and high dietary salt intake, were enrolled as the study population [37, 38].

In this study, UACR was significantly reduced after 8 weeks of dapagliflozin treatment, and this beneficial effect continued until 24 weeks after the start of dapagliflozin treatment, with no reduction in eGFR observed at 24 weeks. This finding suggests that dapagliflozin exerts rapid and sustained renal protective effects on DN, consistent with results from previous studies using SGLT2 inhibitors [10–13, 39–47].

SGLT2 inhibitors are anti-hyperglycemic agents with well-characterized clinical efficacy in the treatment of T2DM. This class of drugs is also reported to exert pleiotropic effects such as decreased body weight, lowered BP, improved arterial stiffness, improvements in nonalcoholic fatty liver disease, and amelioration in endothelial function [9, 11, 14, 15, 39, 43–45, 48–58]. In the current study, glucose-lowering and pleiotropic effects of dapagliflozin were consistently demonstrated [9, 11, 14, 15, 39, 43–45, 48–56]. Although the treatment period with dapagliflozin was only 24 weeks in the present study, short-term improvements in risk markers, especially the albuminuria-lowering properties, with dapagliflozin predict long-term outcomes in patients with T2DM and CKD [59]. The outcomes of several recent clinical trials, such as the EMPA-REG OUTCOME, CANVAS program, and DECLARE-TIMI 58 studies, have indicated the renal protective effects of SGLT2 inhibitors for T2DM patients [10–12]. However, these trials mainly included T2DM patients without DN, and only 10–20% of the patient populations were Asian. Evidence of effects of SGLT2 inhibitors on UACR was limited in Asian patients [60]. More recently, the Canagliflozin and Renal Events in Diabetes with Established Nephropathy Clinical Evaluation (CREDENCE) trial also demonstrated the renal protective effects of a SGLT2 inhibitor for T2DM patients with nephropathy [13]. In the CREDENCE trial, the Asian population was also 19.9%, and out-of-office BPs were not measured. Thus, in the present Y-AIDA study, we focused on Japanese patients with T2DM and DN as the study population. Additionally, we investigated the effects of dapagliflozin on the home BP profile as the key secondary endpoint. Recently, there has been an increasing number of reports about the renal protective effects of SGLT2 inhibitors in T2DM patients with nephropathy [46, 47, 61]. However, there are no reports that have investigated whether SGLT2 inhibitors lower out-of-office BPs in T2DM patients with nephropathy. Additionally, effects of SGLT2 inhibitors on circadian BP patterns, or BP variability, are unknown in T2DM patients with nephropathy.

In the present study, we used a novel home BP measurement device (HEM-7252G-HP) to evaluate the effects of dapagliflozin on out-of-office BP [24]. SGLT2 inhibitors reportedly lower daytime, nocturnal, and 24-h mean BP estimated by ambulatory BP monitoring (ABPM) in hypertensive patients with T2DM [14, 39, 48–52, 62]. However, these studies were of short duration (4–12 weeks), and mainly included T2DM patients without DN. To our knowledge, the present study is the first to demonstrate that add-on dapagliflozin therapy lowers not only morning and evening home BP levels but also nocturnal home BP level over long-term treatment of up to 24 weeks in patients with T2DM and DN. Interestingly, the UACR-reducing effect of dapagliflozin did not correlate with an improvement in glucose metabolism and decrease in body weight or office BP. However, there was a significant correlation between the dapagliflozin-mediated reduction in UACR and the lowering of home mean BP levels. For a causal relationship between reduction in UACR and improvement in the home mean BP levels, it is well known that appropriate blood pressure control can reduce proteinuria in patients with T2DM [7]. Conversely, a decrease in proteinuria could affect blood pressure. It is reported that urinary serine proteases caused by massive proteinuria activate the epithelial sodium channel (ENaC), which leads to increased sodium retention/extracellular fluid and elevated BP in nephrotic syndrome [63, 64]. Although none of the patients in the present study had proteinuria in the nephrotic range, the possibility that dapagliflozin-mediated reduction in albuminuria could lead to a decrease in home BP levels could not be excluded. Additionally, we performed the multivariate linear regression analysis to examine whether the dapagliflozin-induced reduction in the home mean BP levels affected the decrease in UACR. The results showed that the only change in morning home systolic BP was an independent determinant of the decrease in log-UACR. These results suggest that an improved home BP profile contributes to the ameliorating effects of dapagliflozin on albuminuria in T2DM patients with DN.

Asian patients genetically have a high salt sensitivity and a high dietary salt intake [37, 38], and the BP-lowering effects of SGLT2 inhibitors would be a result of their osmotic diuresis and mild natriuresis [39, 65]. Osmotic diuresis might contribute to early decreases in BP by reducing extracellular fluid during SGLT2 inhibitor therapy [66, 67], with natriuresis playing a role in longer term BP reductions [65]. Additionally, dapagliflozin decreases the tissue sodium content of the skin in T2DM patients [68]. Generally, diuretics such as loop and thiazide lower nocturnal BP more than daytime BP [69]. However, in the present study, after add-on dapagliflozin treatment for 24 weeks, the proportion of dippers and extreme-dippers was decreased, and the proportion of non-dippers and risers was increased. These changes might reflect greater reductions in daytime BP compared with those of nocturnal BP. Previous studies also demonstrated that BP reduction with SGLT2 inhibitors persisted throughout a 24-h day with a greater reduction in BP during the daytime compared with nighttime in T2DM patients using ABPM [62]. Kario et al. [70] also reported that empagliflozin treatment for 12 weeks decreased 24-h BP with greater reductions in daytime BP compared with nighttime BP in Japanese patients with T2DM and uncontrolled nocturnal hypertension. This may be partially attributed to the glucose-dependent nature of glycemic control with SGLT2 inhibitors. Osmotic diuresis with SGLT2 inhibitors is likely increased during daytime periods because of higher food and fluid intake. However, urine production during nighttime periods is lower because of circadian rhythms in kidney function. Fluctuation in the sympathetic tone may also affect differences in daytime and nighttime BP regulation with SGLT2 inhibitors.

The current study also showed that add-on dapagliflozin therapy improved the day-by-day variability of home BP, an indicator of quality of BP control, in addition to mean office and home BP levels. Recent studies indicate that the measurement of day-by-day variability of home BP provides a clinically useful distinction between high- and low-CVD risk groups in hypertensive patients [71]. With respect to a possible relationship between BP variability and the progression of renal impairment in T2DM, an increase in day-by-day variability of home BP was reported to be associated with exacerbated albuminuria in T2DM patients with DN [21, 22]. Therefore, the results of the present study indicate that improved BP variability with dapagliflozin partially contributed to the amelioration of DN, and this is the first report to show that SGLT2 inhibitors can not only lower mean BP levels but also improve the quality of BP control.

In the present study, we evaluated urinary biomarkers and oxidative stress markers to explore the mechanism by which dapagliflozin reduces albuminuria in T2DM patients with DN. A decreasing trend was observed for dROMs, an oxidative stress marker, although the change was not statistically significant. Dapagliflozin has been shown to suppress inflammation and oxidative stress in the kidneys of a mouse model of T2DM [72]. In another study with a T2DM mouse model, SGLT2 inhibitors improved albuminuria, glomerular hyperfiltration, and mesangial matrix expansion via an improvement in oxidative stress in the glomerulus [73]. The results of the present study indicate that dapagliflozin may have the potential to reduce oxidative stress in the kidneys of T2DM patients with DN. Some studies reported that improvement in oxidative stress by dapagliflozin contributed to the amelioration of endothelial function in T2DM patients [57, 58]. Sub-clinical inflammation is also involved in the pathogenesis of endothelial dysfunction, which leads to vascular complications including nephropathy in diabetic patients [36]. However, in this study, add-on dapagliflozin therapy for 24 weeks did not change the pro-inflammatory markers such as WBC and Plt. Further studies are therefore required to determine the mechanism responsible for the renal protective action of SGLT2 inhibitors in DN.

The limitations of the present study were as follows. First, the trial design included a single arm, meaning that bias and placebo effects could not be excluded. Second, albuminuria was evaluated through single-spot urine sampling, which may have contributed to large variations in the data. Methods such as 24-h urine collection or multiple spot sampling may provide a more accurate evaluation of albuminuria. Third, this study may be underpowered to estimate the association between the log-UACR decrease and changes in other cardiometabolic parameters because the sample size calculation was based on the expected changes in log-UACR. Fourth, post-prandial glucose levels and continuous glucose monitoring were not measured in this study. It was unknown whether dapagliflozin treatment improved glucose variability, and whether the change in glucose variability affected an improvement in log-UACR.

## Conclusion

Add-on dapagliflozin therapy for 24 weeks improved albuminuria levels as well as out-of-office BP parameters including morning, evening, and nocturnal home BP levels and day-by-day variability of home BP in Japanese patients with T2DM and DN. Additionally, improved morning home systolic BP with dapagliflozin was independently associated with amelioration in albuminuria in this population.

## Additional file

**Additional file 1: Figure S1.** Univariate correlation analysis between the decreases in body weight, body mass index (BMI), fasting blood sugar (FBS), glycated hemoglobin (HbA1c), aspartate aminotransferase (AST), alanine aminotransferase (ALT), alkaline phosphatase (ALP), γ-glutamyl transpeptidase (γ-GTP), white blood cells (WBC), and platelets (Plt), and the decrease in urine albumin-to-creatinine ratio (UACR).

## Data Availability

The datasets are available from the corresponding author on reasonable request and approval by the principal investigator.

## Abbreviations

ABPM: ambulatory BP monitoring
ACE: angiotensin-converting enzyme
AE: adverse event
ALT: alanine aminotransferase
AST: aspartate aminotransferase
ARBs: angiotensin II receptor blockers
BAP: biological antioxidant potential
BMI: body mass index
BP: blood pressure
CK: creatinine kinase
CKD: chronic kidney disease
CVD: cardiovascular diseases
DN: diabetic nephropathy
d-ROMs: diacron-reactive oxygen metabolites
eGFR: estimated glomerular filtration rate
ENaC: epithelial sodium channel
ESRD: end-stage renal disease
FBS: fasting blood sugar
gCr: g creatinine
LDL: low-density lipoprotein
L-FABP: liver-type fatty-acid binding protein
log-UACR: natural logarithm of UACR
MAP: mean arterial pressure
8-OhdG: 8-hydroxy-2′-deoxyguanosine
Plt: platelets
PP: pulse pressure
PR: pulse rate
SD: standard deviation
SE: standard error
SGLT2: sodium–glucose cotransporter-2
T2DM: type 2 diabetes mellitus
UACR: urine albumin-to-creatinine ratio
WBC: white blood cells
