# Supplementary material for: Improved home BP profile with dapagliflozin is associated with amelioration of albuminuria in Japanese patients with diabetic nephropathy: the Yokohama add-on inhibitory efficacy of dapagliflozin on albuminuria in Japanese patients with type 2 diabetes study (Y-AIDA study)
Source: Cardiovasc Diabetol. 2019 Aug 27;18:110. doi: 10.1186/s12933-019-0912-3 (PMC6710883; doi:10.1186/s12933-019-0912-3)
Supplement: Supplementary file 1 — Additional file 1: Figure S1. Univariate correlation analysis between the decreases in body weight, body mass index (BMI), fasting blood sugar (FBS), glycated hemoglobin (HbA1c), aspartate aminotransferase (AST), alanine aminotransferase (ALT), alkaline phosphatase (ALP), γ-glutamyl transpeptidase (γ-GTP), white blood cells (WBC), and platelets (Plt), and the decrease in urine albumin-to-creatinine ratio (UACR). [file 12933_2019_912_MOESM1_ESM.pptx]

## Slide 1
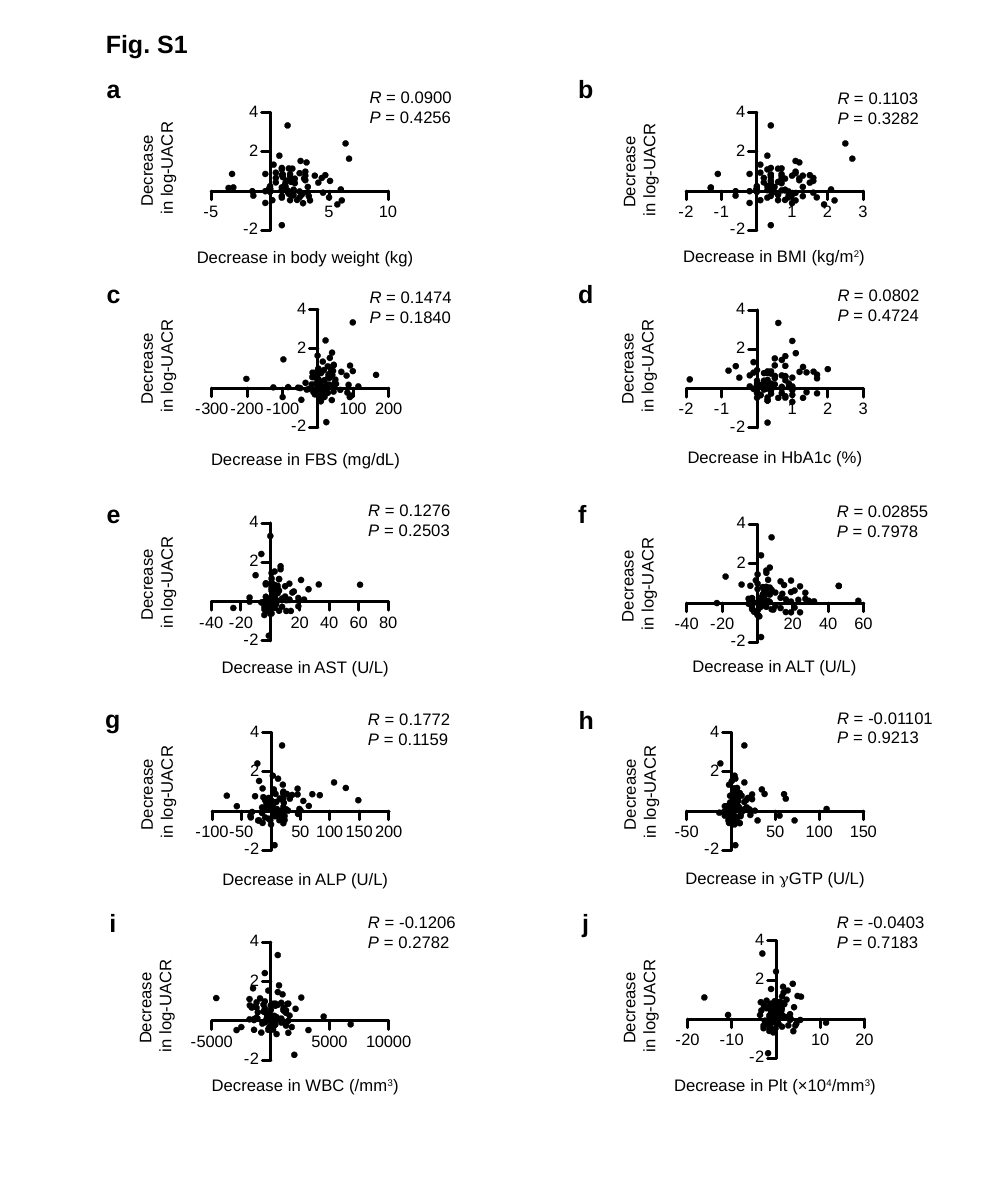

Fig. S1
a
b
R = 0.0900
P = 0.4256
R = 0.1103
P = 0.3282
Decrease
in log-UACR
Decrease
in log-UACR
Decrease in BMI (kg/m2)
Decrease in body weight (kg)
c
d
R = 0.0802
P = 0.4724
R = 0.1474
P = 0.1840
Decrease
in log-UACR
Decrease
in log-UACR
Decrease in HbA1c (%)
Decrease in FBS (mg/dL)
e
f
R = 0.1276
P = 0.2503
R = 0.02855
P = 0.7978
Decrease
in log-UACR
Decrease
in log-UACR
Decrease in ALT (U/L)
Decrease in AST (U/L)
g
h
R = -0.01101
P = 0.9213
R = 0.1772
P = 0.1159
Decrease
in log-UACR
Decrease
in log-UACR
Decrease in gGTP (U/L)
Decrease in ALP (U/L)
i
j
R = -0.1206
P = 0.2782
R = -0.0403
P = 0.7183
Decrease
in log-UACR
Decrease
in log-UACR
Decrease in WBC (/mm3)
Decrease in Plt (×104/mm3)
